# Intraguild Competition between Endangered Kit Foxes and a Novel Predator in a Novel Environment

**DOI:** 10.3390/ani12202727

**Published:** 2022-10-11

**Authors:** Brian L. Cypher, Nicole A. Deatherage, Tory L. Westall, Erica C. Kelly

**Affiliations:** Endangered Species Recovery Program, California State University-Stanislaus, 1 University Circle, Turlock, CA 95382, USA

**Keywords:** competition, endangered species, food habits, habitat attributes, urban environment, *Vulpes macrotis mutica*, *Vulpes vulpes*

## Abstract

**Simple Summary:**

A population of endangered San Joaquin kit foxes inhabits the urban environment in the city of Bakersfield, California, United States. Non-native red foxes, a larger competitor, also occur in Bakersfield and are a potential threat to kit foxes. Based on scat analysis, dietary overlap between the two species is high and red foxes have usurped kit fox dens. Based on logistic regression, habitat attributes generally were similar between grid cells used by each species. However, kit foxes tended to use areas with smaller open spaces and more human activity. We also found that the two species almost never overlapped temporally when using the same grid cells. Because of this temporal separation in addition to an abundance of food and dens in the urban environment, competition from red foxes does not currently appear to be a significant threat to kit foxes.

**Abstract:**

A population of endangered San Joaquin kit foxes inhabits the urban environment in the city of Bakersfield, California, United States. This population is considered important for the conservation and recovery of this species. In this novel environment, kit foxes encounter a novel competitor, that being non-native red foxes. We examined exploitative and interference competition between these two species. Based on scat analysis, both species consumed similar foods and dietary overlap was high. Red foxes also were found to usurp kit fox dens. Direct mortality to kit foxes from red foxes appears to be rare. Kit foxes and red foxes also appear to overlap spatially, although we found evidence of temporal partitioning of shared space. Based on binary logistic regression modeling, habitat attributes in grid cells used by the two species generally were similar, consistent with the spatial overlap. However, differences in specific attributes indicated that kit foxes are more likely to use areas with smaller open spaces and more human activity compared to red foxes. Competition from red foxes may be mitigated by several factors. Critical resources such as food and dens may be sufficiently abundant such that they are not a limiting factor. Some degree of spatial segregation and temporal partitioning of shared space may reduce interference competition. These factors may facilitate coexistence, and consequently, red foxes do not currently appear to constitute a significant competitive risk to this important population of endangered San Joaquin kit foxes.

## 1. Introduction

The San Joaquin kit fox (*Vulpes macrotis mutica*) is a small xeric-adapted canid endemic to arid scrublands and grasslands in the San Joaquin Desert of California, United States [1,2]. The San Joaquin kit fox is listed as Endangered under the U.S. Endangered Species Act and as Threatened under the California Endangered Species Act, primarily due to profound loss and degradation of natural habitat throughout its range [3]. Habitat loss is still occurring. Interestingly, San Joaquin kit foxes occur in the urban environment in the city of Bakersfield, California [4]. This urban population is remarkably robust both demographically and ecologically, and it numbers several hundred individuals [5,6]. This population is considered crucial for the conservation and recovery of San Joaquin kit foxes [5,7].

Red foxes (*Vulpes vulpes*) are widely distributed in California [8]. A red fox subspecies (*V. v. necator*) is native to high elevation montane habitats in the Sierra Nevada and Cascade mountains [9]. However, the red foxes found at lower elevations and throughout much of the state are non-native. These individuals are the descendants of animals, likely from eastern North America, that escaped from fur farms or were introduced for hunting [8,9]. Red foxes are highly adaptable generalists that can use a wide diversity of foods and habitats [10,11]. This facilitated their rapid expansion in California, where they have adversely impacted rare species through predation, including the California clapper rail (*Rallus longirostris obsoletus*), lightfooted clapper rail (*R. l. levipes*), and California least tern (*Sterna antillarum browni)* [8,12].

Red foxes also potentially adversely impact San Joaquin kit foxes (hereafter kit foxes) [13]. In intraguild interactions among carnivores, larger species typically are competitively dominant over smaller species [14], and this holds true for canids. Among North American fox species, red foxes are the largest [11], and kit fox deaths have been attributed to red foxes [15,16]. In addition, in the San Joaquin Desert, dietary overlap between kit foxes and red foxes can be high, and red foxes may usurp kit fox dens [16]. Fortunately, red foxes are uncommon in natural habitats in this region, likely due to intense predation pressure from coyotes (*Canis latrans*) [13]. Whereas kit foxes elude coyotes through den use [17], red foxes rely on dense cover but cover tends to be sparse in the arid habitats of the San Joaquin Desert [2]. Thus, red foxes primarily occur in anthropogenically disturbed areas, such as agricultural lands and urban environments, where coyotes are less common. San Joaquin kit foxes rarely use agricultural lands [18] but do occur in certain urban areas. 

In the city of Bakersfield, San Joaquin kit foxes are abundant as described previously, and red foxes are regularly observed as well [5,19]. Thus, kit foxes have occupied a novel environment and are encountering a novel, non-native competitor that they rarely encounter in natural habitats. The effects of red foxes on this important kit fox population are unknown. We assessed the potential for interference and exploitative competition by red foxes on San Joaquin kit foxes in Bakersfield. Specific objectives were to (1) compare food use and dietary overlap by kit foxes and red foxes, (2) assess den use of urban red foxes, (3) assess landscape-scale spatial overlap, and (4) compare habitat attributes between areas used by kit foxes and red foxes.

## 2. Materials and Methods

### 2.1. Study Area

**Figure 1:**
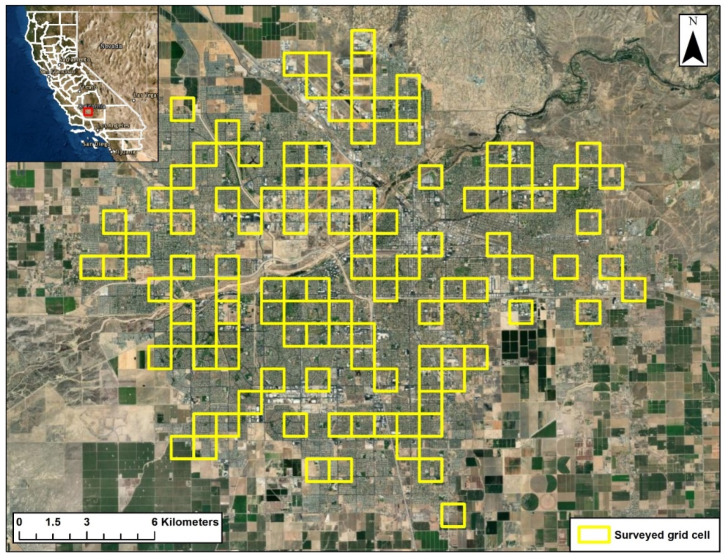
City of Bakersfield, California, with 1 km^2^ grid cells that were surveyed for the presence of San Joaquin kit foxes and red foxes from 2015 to 2021.

This study was conducted in the city of Bakersfield, which is located in Kern County in the southern San Joaquin Valley in central California, USA (Figure 1). As of 2020, the city had an area of 388 km^2^ and a human population of ca. 391,438 [20]. Average elevation is 124 m, with little topographic variation. Climate is characterized by hot, dry summers and cool winters with infrequent precipitation in the form of rain. Average high and low temperatures are 13.7 °C and 3.9 °C in December and 36.2 °C and 21.4 °C in July. Mean annual precipitation is 164 mm [21]. Bakersfield is bounded by occupied kit fox habitat to the northeast and southwest (1) with irrigated agriculture bordering the city elsewhere.

### 2.2. Food Item Use

Food item use by kit foxes red foxes was assessed through analysis of scat (fecal) samples. Kit fox and red fox scats overlap considerably in size and appearance. Thus, kit fox scats were collected from live-traps (55%) in which kit foxes were captured (for other studies) and from around dens (45%) being used by kit foxes. Red fox scats were collected from around dens being used by red foxes. Scats were placed in paper bags labeled with the date and coordinates for the location. Scats were oven-dried at 60 °C for ≥24 h to kill any parasite eggs and cysts. The scats then were placed in individual nylon bags, washed to remove soluble materials, and then dried in a tumble dryer. Remaining undigested material was examined to identify food items. Mammalian remains (e.g., hair, teeth, bones) were identified using macroscopic (e.g., length, texture, color, banding patterns) and microscopic (e.g., cuticular scale patterns) characteristics of hairs [22] and by comparing teeth and bones to reference guides [23,24] and specimens. Other vertebrates were identified to class and invertebrates to order, based on feathers, scales, and exoskeleton characteristics and comparison to reference specimens. 

Annual frequency of occurrence of items in scats was determined for kit foxes and red foxes. Items also were grouped into one of five broader categories: mammal, bird, reptile, invertebrate, and anthropogenic items. Occurrence frequencies for this categorical array of items was compared between kit foxes and red foxes using contingency table analysis and an χ^2^ statistic [25]. Frequencies of occurrence for each individual category were compared between kit foxes and red foxes using a 2 × 2 contingency table analysis and an χ^2^ statistic employing Yate’s correction for continuity [25]. Finally, we calculated a Shannon diversity index for categorical item arrays for both kit foxes and red foxes and also calculated a Horn’s similarity index to examine dietary overlap between the two species [26].

### 2.3. Red Fox Den Use

Ideally, red fox dens would have been located by tracking radio-collared animals. However, we did not have that opportunity in this study. Thus, observations of red foxes at dens were collected opportunistically, either by our field staff or when reported by other biologists working in Bakersfield. Red foxes primarily use dens during the breeding season. Thus, den use is not frequent. Red foxes also generally use dens in denser cover where they are not easily discovered. When a red fox was observed at a den, the location was compared with a database on kit fox den locations collected during an on-going demographic and ecological investigation on urban kit foxes [5]. We attempted to determine whether any of the dens used by red foxes had previously been used by kit foxes.

### 2.4. Spatial Overlap

Sarcoptic mange was observed in kit foxes in Bakersfield in 2013 and spread rapidly throughout the Bakersfield kit fox population [27]. In an effort to assess the extent of mange spread and to monitor the population response by kit foxes, we conducted annual surveys from 2015 to 2021 for kit foxes and other species using camera stations. We layered a sampling grid consisting of 357 1 km^2^ grid cells over aerial imagery of the city. We used the randomization function in Excel 2010 (Microsoft, Redmond, WA, USA) to randomly choose 120 grid cells to potentially sample from the 357 available cells. Within each selected grid cell, we then identified locations (1) that were accessible to kit foxes and (2) where the risk of camera theft was low (i.e., locations with restricted public access or where a camera could be placed in a cryptic location). Consequently, most camera stations were placed in locations such as school campuses, city or county storm water drainage basins, municipal facilities, churches, golf courses, private businesses (with owner permission), and undeveloped parcels. 

Within each sampled grid cell, we employed an automated camera station design and methodology developed specifically to survey for kit foxes and other sympatric carnivores [28]. One station was established in each sampled cell and operated for one week. We used Cuddeback Digital Black Flash IR cameras (Model 1255, Non Typical Inc., Green Bay, WI, USA) that employ a “black flash” infrared LED flash that creates almost no light visible to humans and that take high-resolution images (20 megapixels). We secured the cameras to 1.2 m U-posts using zip-ties. At some locations where cameras might be discovered by the public, we placed the cameras in protective cases (“CuddeSafe” Model 3327, Non Typical Inc., Green Bay, WI, USA) that were then secured with a cable lock to fences, trees or other immobile structures. To attract foxes, we placed approximately 5 drops of a scent lure (Carman’s Canine Call Lure, New Milford, PA, USA) approximately 2 m in front of the camera and on surrounding vegetation. We also staked a 163 mL can of cat food to the ground approximately 2 m in front of each camera using 30 cm nails. The cat food cans were perforated to allow scent to void but limit access to the food. The staked cat food cans functioned as a further attractant for foxes and also caused them to remain in the camera’s field of view for an extended period as foxes attempted to access the food in the cans.

At the end of one week, the cameras were collected and the captured images were examined for the presence of kit foxes, red foxes, and other species. The grid cells in which either kit foxes or red foxes were detected were recorded as well as the number of years that each species was detected in each cell. We also recorded the number of stations that were visited by both species in a given year. Finally, we recorded the number of nights that stations were visited by each species and the number of nights that both species visited a given station. Proportions of grid cells, annual detections at a given station, and station-nights where both kit foxes and red foxes were detected were compared to those where only red foxes were detected using *z* tests.

### 2.5. Habitat Attributes

We used satellite imagery to quantify seven urban landscape attributes in each of the sampled cells. The attributes were identified by Deatherage et al. [29] as being important for urban kit foxes and are described in Table 1. To quantify the attributes, we superimposed a point grid (10 × 10 grid, 100 points total) over each cell in Google Earth Pro. We used Google Earth Pro imagery dated 26 April 2018 at an eye altitude of 300 m above ground level to characterize grid points. We characterized each point by the attribute that best described the location of the point (i.e., the terrestrial land use type on which the majority of the point was located or closest to). If a point appeared to fall equally on two different landscape types, we split the proportion of the point equally between the attributes (0.5:0.5). The proportion of points in each land use category in a given cell was considered to approximate the proportion of that cell consisting of that land use. 

To normalize the data, an arcsine transformation was applied to the proportional estimates for the land use categories for each cell [25]. We tested for pairwise correlations between landscape attributes using Spearman’s Rank tests. No attribute pair had a correlation coefficient greater than 0.7, and therefore no attributes were excluded from candidate explanatory models. We used multi-variate binary logistic regression on the transformed variables to assess the relationship between landscape attributes and visits to camera stations by kit foxes and red foxes. Candidate models were generated consisting of all possible combinations of variables. Akaike’s Information Criterion for small sample sizes (AIC_c_) was used to rank models and determine the combinations of attributes and the individual attributes that best explained which cells had detections of kit foxes or red foxes at the camera stations. AIC methods followed Burnham and Anderson [30]. Models were ranked based on ΔAIC_c_ values and strength of each model was evaluated based on Akaike weights. Models with Akaike weights within 10% of the highest weight were included in the confidence set of candidate models [30,31]. The relative importance of each in the confidence set of candidate models was evaluated by summing the Akaike weights of the models that included the attribute. Finally, two-tailed Student *t*-tests were used to compare mean transformed values for attributes between cells with kit fox detections and cells with red fox detections. For each species, the attribute values in a given cell were weighted based on the number of years that the species was detected in the cell. For example, if a kit fox was detected in a given cell in four different years, the values for that cell were included as four observations in the kit fox data set. An α-level of 0.05 was used to determine statistical significance.

## 3. Results

### 3.1. Food Item Use

We analyzed 720 kit fox scats and 763 red fox scats that were collected during 2000–2010. Both species consumed a diversity of food items (Table 2). At least 12 different items were found in kit fox scats, and at least 17 different items were found in red fox scats. Items of particular importance to kit foxes included rodents (particularly California ground squirrels (*Otospermophilus beechyi*)), birds (specific species rarely could be identified), invertebrates (particularly beetles (Coleoptera)), and anthropogenic materials (based on the presence of plastic wrap, aluminum foil, and other food wrapping materials in the scats). Items of particular importance to red foxes included rodents (particularly pocket gophers (*Thomomys bottae*) and California ground squirrels), birds, invertebrates (particularly beetles), and anthropogenic materials. 

**Figure 2:**
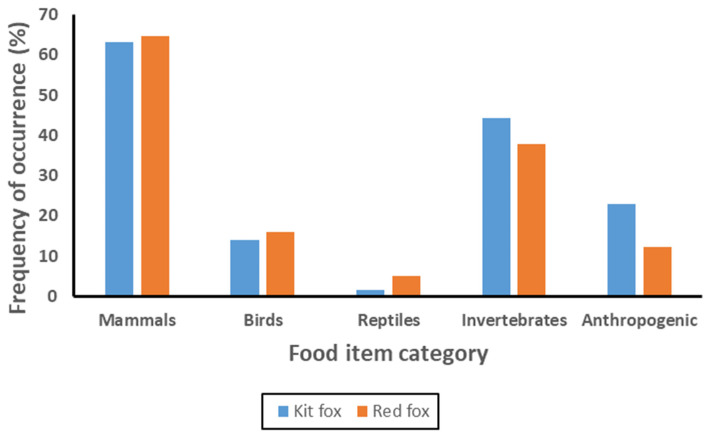
Frequency of occurrence of food items by category in scats of San Joaquin kit foxes and red foxes in Bakersfield, California, during 2000–2010.

When grouped into broader categories (Figure 2), frequency of occurrence for the array of categories differed between kit foxes and red foxes (χ^2^ = 39.45, 4 df, *p* < 0.001). For individual categories, frequency of occurrence differed between kit foxes and red foxes for reptiles (χ^2^ = 12.22, 1 df, *p* < 0.001), invertebrates (χ^2^ = 6.06, 1 df, *p* = 0.014), and anthropogenic materials (χ^2^ = 28.92, 1 df, *p* < 0.001), but not for mammals (χ^2^ = 0.39, 1 df, *p* = 0.532) or birds (χ^2^ = 0.97, 1 df, *p* = 0.325). Despite these differences, dietary diversity based on the categories was similar between kit foxes (0.561) and red foxes (0.565), and Horn’s similarity index was 0.986, indicating that dietary overlap was high. 

### 3.2. Red Fox Den Use

Only 10 dens used by red foxes were identified in Bakersfield. However, at least 9 of these dens were known to have been used by kit foxes. 

### 3.3. Spatial Overlap

**Figure 3:**
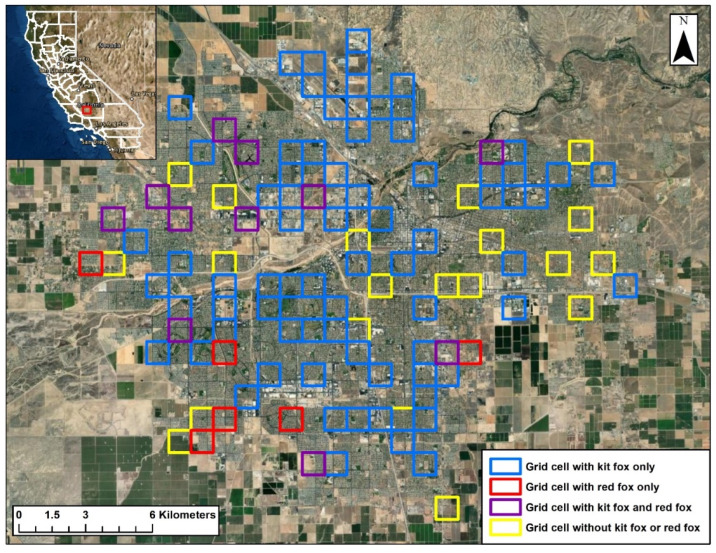
Grid cells in the Bakersfield, California, where San Joaquin kit foxes, red foxes or both species were detected during 2015–2021.

From 2015–2021, camera station surveys were conducted in 112 of the grid cells each year. Kit foxes were detected in 87 of the cells with detections ranging from 23 to 68 cells per year (Figure 3). Red foxes were detected in 17 of the cells, with detections ranging from 4 to 8 cells per year. Kit foxes were detected in 11 of the 17 cells that red foxes were detected in. The proportion of cells used by red foxes that were also used by kit foxes (64.7%) was marginally higher than the proportion used by red foxes only (35.3%; *z* = 1.7, *p* = 0.087). However, of the 33 annual red fox detections in specific cells during the 7-year study period, kit foxes were detected in those same cells in the same year as red foxes on only 3 occasions (9.1%), which was significantly less (*z* = 6.6, *p* < 0.001) than proportion of occasions when only red foxes were detected (90.9%). Furthermore, red foxes were detected on 71 camera-nights during the study, and kit foxes were only detected 1 time at a station on the same night that a red fox was detected. The proportion that both species were detected on the same night (1.4%) was significantly lower (*z* = 11.6, *p* < 0.001) than the proportion of nights that only red foxes were detected (98.6%).

### 3.4. Habitat Attributes

The relationship between kit fox or red fox presence in a given cell and habitat attributes in that cell was assessed for the 92 cells in which one or both species were detected (Figure 3). All combinations of the seven habitat attributes measured were used to generate 83 candidate binary logistic regression models. Using ΔAIC_c_ values to rank the models and Akaike weights to evaluate the potential explanatory strength of each model, five models were included in the confidence set of candidate models (Table 3). The top model included all of the habitat attributes and an Akaike weight of 0.3644. Thus, none of the models had particularly strong explanatory value. Based on the Akaike weights, the proportions of residential, undeveloped, commercial, and industrial areas had equally high importance weights (Table 4) followed by the proportions of campus, park, and road areas. For the top model, β-values were significant or near significant for all parameters except roads and the proportion of commercial areas (Table 5). Finally, the number of annual detections in cells (number of years that a species was detected in a given cell summed across all cells) totaled 263 across 87 cells for kit foxes and 32 across 17 cells for red foxes. This resulted in 295 observations for comparing mean attribute values in cells used by two species. The mean proportion of campus areas was significantly higher in cells used by kit foxes, and the proportion of commercial areas was marginally higher (Table 6).

## 4. Discussion

Based on our results, red foxes potentially engage in competitive interactions with San Joaquin kit foxes in the urban environment of Bakersfield, California. The intensity of this competition varies across the urban landscape and among years. This variation creates opportunities for spatial and temporal segregation. Such segregation may be sufficient to reduce competitive pressure on kit foxes and facilitate coexistence between the two species.

Kit foxes and red foxes overlap extensively in use of food items, although several differences in use of specific food categories were detected. Red foxes consumed more lizards and snakes. These items may be more abundant in the larger, less developed areas that red foxes tend to inhabit (see below). Kit foxes consumed more invertebrates. Kit foxes are only 30–50% the size of red foxes [11] and therefore are able to obtain adequate nutrition from smaller items. Similarly, corsac foxes (*V. corsac*), which are similar in size than kit foxes, also consumed more invertebrates relative to sympatric red foxes in Mongolia [32]. Kit foxes also consumed anthropogenic items more frequently than red foxes. This may result from kit foxes generally using areas with more human activity, such as campuses and commercial areas (see below). Red fox food-item use also can overlap extensively with that of arctic foxes in arctic regions, resulting in decreased arctic fox abundance, especially during periods of low prey availability when exploitative competition intensifies [33,34]. Similarly, overlap in food habits between corsac foxes and red foxes in Mongolia increased in winter when food availability was lower, and exploitative competition between the two species potentially increased as well [32]. In Bakersfield, food resources are super-abundant due to the presence of anthropogenic food items (e.g., trash, accidentally dropped food, pet food left outdoors, intentional feeding of foxes) in addition to natural food items. Thus, despite high dietary overlap, exploitative competition between kit foxes and red foxes may be weak if food item availability is not a limiting factor.

Red foxes commonly usurp artic fox (*Alopex lagopus*) dens where the two species are sympatric [35,36]. Red foxes also clearly usurp kit fox dens in the urban environment. Almost all of the dens that we found being used by red foxes were known to have been previously used by kit foxes. In addition, in a study conducted in Bakersfield to determine the value of artificial dens in kit fox conservation efforts, red foxes were detected using 5 of the 31 dens, all of which also had also been used by kit foxes [37]. Red foxes also were observed to have usurped known kit fox dens at Camp Roberts, a California Army National Guard Training Site in Central California [38]. Dens are another resource for which kit foxes and red foxes potentially compete. However, as with food resources, dens do not appear to be a limiting factor for kit foxes in Bakersfield [5]. Each kit fox commonly has about a dozen dens within its home range and losing access to one den is unlikely to be problematic. In addition, red fox abundance is relatively low compared to that of kit foxes [19]. Lastly, red foxes primarily use dens during a limited period of time, that being in the late spring or early summer during pup-rearing. Thus, occasional use of kit fox dens by red foxes is not likely to adversely impact the kit fox population in Bakersfield.

Patterns that we found in space use and habitat attributes between kit foxes and red foxes could be a function of interference competition (i.e., kit foxes avoiding red foxes), differences in habitat preferences or a combination of these. Kit foxes were detected using most of the grid cells used by red foxes. However, only uncommonly (9.1% of occasions) were the two species detected in the same grid cell in the same year. Furthermore, only once were kit foxes and red foxes detected at the same station on the same night. Interestingly, kit foxes were observed at that station for six consecutive nights, a red fox then was detected on the sixth night, but kit foxes were not detected at that station after that night. Based on visits to camera stations, spatial overlap between kit foxes and red foxes is high, but kit foxes appear to exhibit temporal avoidance of areas actively being used by red foxes. Thus, some degree of interference competition may be occurring.

The analyses of habitat attributes used by kit foxes and red foxes also provided evidence for spatial overlap between the two species. The top models in our logistic regression analyses contained all or most of the habitat attributes, and parameter weights did not readily distinguish particular attributes as being more important in explaining observed use of grid cells by the two species (see Table 3 and Table 4). Our inability to identify models and parameters with strong explanatory power actually is consistent with the extensive overlap in use of common grid cells by kit foxes and red foxes. Clearly, the types of areas used by the two species are generally similar. The comparison of mean attributes did reveal some minor differences. The mean proportion of campus area was significantly higher for kit foxes and the mean proportion of commercial area was marginally higher. In both cases, the differences between the means was not great but was sufficient to achieve statistical significance. If attribute use could have been examined on a finer scale (e.g., by tracking radio-collared individuals), then differences in mean attributes might have been more pronounced. However, the results were consistent with observations and past analyses [5,6,29], indicating that kit foxes are frequently observed on campuses, particularly college and K-12 school campuses, and in the vicinity of commercial areas (e.g., retail stores, restaurants, office complexes). Anthropogenic food availability generally is high in these areas. In addition, kit foxes frequently establish dens under the portable buildings that are ubiquitous on campuses all throughout Bakersfield. Red foxes in Bakersfield tend to occur in areas with large open spaces and less human activity. Such areas include the extensive fields on the California State University-Bakersfield campus, canal and railroad rights-of-way, and large undeveloped lots. 

Another form of interference competition is agonistic encounters that can result in injury or death, usually to the smaller of the combatants. The literature is replete with examples among mammalian carnivores [39]. However, such interactions involving two fox species and any resulting adverse population effects have not been well-documented. Red foxes have been documented killing corsac foxes [40]. Agonistic interactions have best been documented between red foxes and arctic foxes. Red foxes have frequently been documented killing arctic foxes, sometimes to the point of reducing arctic fox abundance or limiting their distribution [41,42,43,44]. Very interestingly, red foxes also essentially constitute novel competitors for arctic foxes. Due to climate change, habitat change, reductions of larger predators such as wolves (*C. lupus*), and other factors, red foxes are expanding their range into artic and alpine environments that used to be the exclusive domain of arctic foxes [35,41,45,46]. 

Similarly, red foxes have expanded into the range of the San Joaquin kit fox. As with arctic foxes, kit fox mortalities attributable to red foxes have been documented [15,16]. Indeed, kit fox hair was found in a red fox scat in Bakersfield, although it is unknown whether this represents predation or scavenging. However, as detailed previously, red foxes are quite rare in natural habitats within the range of the San Joaquin kit fox. In the urban environment of Bakersfield, red fox distribution appears to be limited to areas with certain favorable conditions (e.g., large open spaces, lower levels of human activity). In a study conducted in Bakersfield from 1997 to 2004, 78 of 229 radio-collared kit foxes were recovered dead, and none of the mortalities were definitively attributed to red foxes [47]. Thus, interference competition in the form of agonistic encounters does not appear to be a significant factor for urban kit foxes. 

The extent to which red foxes may harass or chase kit foxes resulting in exclusion from specific locations is unknown. We suspect that this likely occurs on occasion. However, kit foxes apparently will also “stand their ground” against red foxes. Westall et al. [48] reported a male kit fox exhibiting aggressive behavior toward a red fox that had approached a kit fox natal den. Similar behavior was observed by kit foxes at another natal den that actually was located only about 100 m from a red fox natal den. Thus, red fox presence clearly does not always discourage use of an area by kit foxes.

Urban habitats constitute a novel environment for San Joaquin kit foxes, and non-native red foxes constitute a novel competitor. Based on overlap in food item use, space use, and habitat attributes, red foxes potentially engage in competitive interactions with kit foxes inhabiting Bakersfield, California. Red foxes have been documented to adversely impact other small fox species. However, to date, the Bakersfield kit fox population does not appear to be adversely affected by red foxes. The reasons for this may be multiple. Critical resources such as food and dens may be sufficiently abundant such that they are not a limiting factor, and therefore competition for these resources between the two species may be weak at best. Direct mortality to urban kit foxes from red foxes also appears to be rare. Some differences in habitat preferences may result in some degree of spatial segregation between kit foxes and red foxes, and where they do overlap kit foxes exhibit apparent temporal partitioning to avoid red foxes. Furthermore, red foxes have a more limited distribution in the urban environment compared to kit foxes, based on our camera survey data. Finally, as further evidence that red foxes do not appear to be a limiting factor for kit foxes, in the absence of mange, kit fox density in the urban environment is considerably higher than that in non-urban environments [6].

## 5. Conclusions

The scientific and popular literature are replete with abundant examples of non-native species impacting populations of rare species. This situation is exacerbated for rare species that also are subject to other threats such as habitat loss, disease, accidental or illegal mortality or pollution. San Joaquin kit foxes are threatened by habitat loss, past exploitation, accidental deaths from predator control programs [3], and more recently sarcoptic mange. Thus, the presence of non-native red foxes, a larger competitor, potentially further increases the risk of extinction for kit foxes. Fortunately, red foxes are sympatric with San Joaquin kit foxes primarily in urbanized environments. In these environments, critical resources such as food and den sites appear to be sufficiently abundant to mitigate competition from red foxes. Kit foxes also appear to exhibit some degree of spatial and temporal partitioning that further reduces competition. Consequently, red foxes currently do not appear to constitute a significant competitive risk to the important population of San Joaquin kit foxes present in the urban environment of Bakersfield.

## Figures and Tables

**Table 1:** Urban landscape attributes quantified in 1 km^2^ grid cells surveyed annually for San Joaquin kit foxes and red foxes in Bakersfield, California, United States, from 2015 to 2019.

Attribute	Description
*Road*	Any paved public road carrying vehicular traffic
*Res*	Residential areas including single-family and multi-family housing
*Und*	Undeveloped parcels of various sizes within the urban landscape
*Ind*	Industrial areas including refineries, manufacturing facilities, and pipe and equipment storage yards
*Park*	Parks and green spaces such as city parks, recreational areas, golf courses, and cemeteries
*Camp*	Campuses including schools, churches, and large medical centers
*Com*	Commercial areas including office buildings, hotels, shopping centers, restaurants, and other businesses

**Table 2:** Frequency of occurrence of items in scats from urban kit foxes and red foxes in Bakersfield, California, during 2000–2010.

Item	Frequency of Occurrence (%)	
	Kit Foxes (n = 720 Scats)	Red Foxes (n = 763 Scats)
Mammals		
Pocket gopher	7.4	31.9
California ground squirrel	10.7	21.9
Deer mouse	0.3	-
Rat	-	0.1
Kangaroo rat	-	0.1
Pocket mouse	-	0.3
Leporid	2.2	3.4
Cat	-	0.1
Kit fox	-	0.1
Birds		
Unidentified bird	14.0	16.0
Reptiles		
Unidentified lizard	0.3	0.7
Unidentified snake	0.7	3.9
Invertebrates		
Jerusalem cricket	-	0.1
Grasshopper	0.3	-
Cricket	-	0.4
Cockroach	1.8	-
Beetle	13.1	26.2
Arachnid	0.3	0.4
Solpugid	-	0.1
Anthropogenic materials		
Food wrappers	22.9	12.2

**Table 3:** Confidence set of candidate models and associated Akaike Information Criterion values for binary logistic regression analysis of the relationship between habitat attributes and visits to camera stations by San Joaquin kit foxes or red foxes in Bakersfield, California, during 2015–2021.

Model	Model ^1^	−2LL ^2^	K ^3^	AICc ^4^	ΔAICc	Wi ^5^
1	Troad + Tres + Tund + Tcom + Tind + Tcamp + Tpark	170.245	9	188.8766	0	0.3644
2	Tres + Tund + Tcom + Tind + Tcamp + Tpark	172.919	8	189.4225	0.5459	0.2773
3	Troad + Tres + Tund + Tcom + Tind + Tpark	174.489	8	190.9925	2.1159	0.1265
4	Tres + Tund + Tcom + Tind + Tcamp	177.718	7	192.1082	3.2317	0.0724
5	Troad + Tres + Tund + Tcom + Tind + Tcamp	176.975	8	193.4785	4.6019	0.0365

^1^ Model variables: *road* = proportion of roads, *res* = proportion of residential area, *und* = proportion of undeveloped area, *com* = proportion of commercial area, *ind* = proportion of industrial area, *camp* = proportion of campus area, and *park* = proportion of parks and other green space area. “*T*” indicates that all variables were transformed prior to analysis using an arcsine transformation. ^2^ −2*log likelihood. ^3^ Number of parameters in the model. ^4^ Akaike’s Information Criterion value for small sample sizes. ^5^ Akaike model weight.

**Table 4:** Importance weights of parameters in the confidence set of candidate models for binary logistic regression analysis of the relationship between habitat attributes and visits to camera stations by San Joaquin kit foxes or red foxes in Bakersfield, California, during 2015–2021.

	Parameter Importance Weights						
	Troad	Tres	Tund	Tcom	Tind	Tcamp	Tpark
Model 1	0.3644	0.3644	0.3644	0.3644	0.3644	0.3644	0.3644
Model 2	0	0.2773	0.2773	0.2773	0.2773	0.2773	0.2773
Model 3	0.1265	0.1265	0.1265	0.1265	0.1265	0	0.1265
Model 4	0	0.0724	0.0724	0.0724	0.0724	0.0724	0
Model 5	0.0365	0.0365	0.0365	0.0365	0.0365	0.0365	0
Total weight	0.5274	0.8771	0.8771	0.8771	0.8771	0.7506	0.7682

**Table 5:** Parameter values for the top binary logistic regression model of the relationship between habitat attributes and visits to camera stations by San Joaquin kit foxes or red foxes in Bakersfield, California, during 2015–2021.

Parameter	β	S.E.	Wald	df	*p*	Exp(β)
*Troad*	4.040	2.537	2.535	1	0.111	56.815
*Tres*	7.164	2.083	11.832	1	<0.01	1291.714
*Tund*	6.380	2.137	8.911	1	0.003	589.966
*Tcom*	−2.038	1.895	1.157	1	0.282	0.130
*Tind*	6.807	2.161	9.918	1	0.002	903.782
*Tcamp*	−3.395	1.754	3.747	1	0.053	0.034
*Tpark*	5.109	2.014	6.435	1	0.011	165.454
*Intercept*	−12.630	3.772	11.209	1	<0.01	0.000

**Table 6:** Comparison of mean habitat attribute values between cells used by San Joaquin kit foxes and red foxes in Bakersfield, California, during 2015–2021.

	% Cell Composition				
Attribute	Kit Fox	Red Fox	t	df	*p*
Road	21.0	19.1	−0.933	293	0.352
Residential	37.5	41.9	0.952	293	0.342
Undeveloped	19.4	20.4	0.250	293	0.803
Commercial	5.0	2.8	−1.623	293	0.106
Industrial	6.3	8.9	1.207	293	0.229
Campus	5.2	1.9	−2.563	293	0.011
Park/green space	3.6	4.1	0.472	293	0.637

## Data Availability

Data collected during this study are available upon reasonable request.
